# Does gamma-glutamyltransferase correlate with liver tumor burden in neuroendocrine tumors?

**DOI:** 10.1007/s12020-023-03545-x

**Published:** 2023-09-28

**Authors:** Benjamin Christopher Schmidt, Miriam Theresa Leiderer, Tania Amin, Fabrice Viol, Samuel Huber, Frank Oliver Henes, Jörg Schrader

**Affiliations:** 1 Department of Medicine I, University Medical Center Hamburg-Eppendorf, Hamburg, Germany; 2 Department of Medicine II, University Medical Center Hamburg-Eppendorf, Hamburg, Germany; 3 Department of Diagnostic and Interventional Radiology and Nuclear Medicine, University Medical Center Hamburg-Eppendorf, Hamburg, Germany; 4 Department of Medicine, Klinikum Nordfriesland, Husum, Germany

**Keywords:** Neuroendocrine tumor, Gamma-glutamyltransferase, Liver tumor burden, Therapy monitoring

## Abstract

**Purpose:**

In patients with neuroendocrine tumors (NETs) and liver metastases, increased gamma-glutamyltransferase (GGT) is commonly assumed as an indicator for progressive disease. To date, however, empirical data are lacking. This study aimed to investigate associations between GGT and liver tumor burden. In longitudinal analyses, associations of GGT and radiographic responses of liver metastases under therapy were investigated.

**Methods:**

The cross-sectional sample consisted of 104 patients who were treated at the University Medical Center Hamburg-Eppendorf from 2008 to 2021 (mean age 62.3 ± 12.6 years, 58.7% male). GGT and liver imaging were identified in a time range of 3 months. Radiologic reassessments were performed to estimate liver tumor burden. In a separate longitudinal sample (*n* = 15), the course of GGT levels under chemotherapy was analyzed. Data were retrospectively analyzed with a univariate ANOVA, linear regression analyses, and Wilcoxon tests.

**Results:**

Of 104 cross-sectionally analyzed patients, 54 (51.9%) showed a GGT elevation. GGT levels and liver tumor burden were positively correlated (*p* < 0.001), independently from age, gender, primary tumor location, grading, and cholestasis. Notably, GGT increase was associated with a liver tumor burden of >50%. In the longitudinal sample, 10 of 11 patients with progressive disease showed increasing GGT, whereas 4 of 4 patients with regressive disease showed declining GGT.

**Conclusion:**

Our findings indicate that GGT is associated with liver tumor burden. Over the course of therapy, GGT appears to change in line with radiographic responses. Further longitudinal studies with larger sample sizes are required to define GGT as a reliable marker for tumor response.

## Introduction

In metastatic neuroendocrine tumors (NETs), liver metastases are present in 82% of patients [1]. In addition to surgical therapy, systemic treatment is often required to maintain the quality of life and prevent unhindered progression of the tumor disease [2]. Over the course of systemic treatment, response is usually monitored by radiographic imaging. Since regular scans are necessary, this approach strains healthcare resources and is often responsible for high radiation exposure to the patient. Laboratory markers could provide an additional tool for therapy monitoring, reducing the frequency of imaging.

Gamma-glutamyltransferase (GGT) is a membrane-bound glycoprotein and a key enzyme of the gamma-glutamyl cycle. It is required for the transport of amino acids across the membrane and in particular for the provision of glutathione, one of the most important antioxidants of the human body [3]. It is found mainly in epithelia with high secretory or absorptive functions, such as renal tubules, bile ducts, liver, pancreas, and intestine [3]. It has been used as a laboratory marker for more than 50 years and is considered to be one of the most sensitive biomarkers for liver conditions in general [4]. Serum GGT is associated with increased oxidative stress [5]. Typical clinical conditions in which GGT is elevated are alcohol consumption, cholestasis, or drug intake. However, in patients with malignancies, elevated GGT can also be a sign of advanced disease [6, 7]. It is associated with poor prognosis in patients with hepatocellular carcinoma [8], renal cell carcinoma [9], ovarian [6], and endometrial cancer [10]. In clinical follow-up of NETs, increased GGT levels are especially seen in patients with liver metastases. To the best of our knowledge, there is no published data on the prevalence of increased GGT levels in patients with NETs and possible associations with liver tumor burden.

**Fig. 1:**
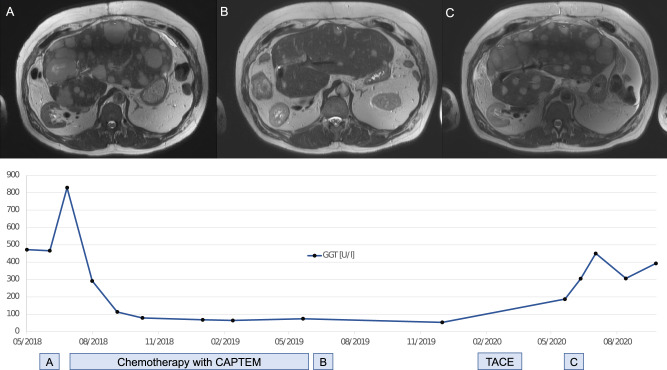
Exemplary course of gamma-glutamyltransferase and liver tumor burden. Notes: Images **A**, **B** and **C** were captured in the same layer of transverse post contrast T1-weighted magnetic resonance imaging. GGT gamma-glutamyltransferase, CAPTEM Capecitabine/Temozolomide, TACE transarterial chemoembolization

To exemplify the clinical association between GGT and liver tumor burden, we report the case of a patient with pancreatic NET G2 (male, 40 years old). Upon the initial diagnosis in 2004, he underwent a partial pancreatectomy and splenectomy. After a long and stable treatment course with Lanreotide from 2005 to 2018, laboratory testing showed significantly increased GGT. In an abdominal magnetic resonance imaging (MRI), a distinct hepatic progression was found (Fig. 1A). Another biopsy of the tumor was performed, revealing a grade progression from G2 to G3. Consecutively, the therapy regimen was changed to oral chemotherapy with Capecitabine/Temozolomide (CAPTEM). Hereunder, partial remission with high response of liver metastases was achieved, while GGT decreased from an over tenfold increase of the upper normal limit back to normal values (Fig. 1B). In early 2020, routine imaging showed stable disease but growth of two liver metastases. Transarterial chemoembolization was performed. In the next laboratory follow-up, GGT levels increased noticeably. Abdominal MRI showed another hepatic progression (Fig. 1C). Third-line therapy was initiated within a clinical study. In this case report, GGT changes were documented twice before routine imaging, so serial GGT testing helped to detect progression and lead to adaptation of therapy. However, analyses of a larger sample are necessary to extrapolate these individual findings to clinical practice.

This study primarily aimed to investigate associations between GGT and liver tumor burden in a cross-sectional sample of NET patients with liver metastases. We hypothesized that high GGT values are associated with high liver tumor burden. To evaluate the impact of GGT in predicting the clinical course under therapy, we secondarily analyzed a separate small sample of patients undergoing Streptozotocin/5-Fluorouracil (STZ/5FU) treatment for pancreatic NET. This sample was chosen since the clinical course was well documented and GGT levels were available for each cycle of therapy. In addition, this therapy holds the potential for partial remission [11], thus giving the opportunity to demonstrate GGT dynamics in case of decreasing tumor burden. We hypothesized that progression or regression of liver metastases are associated with GGT increase or decrease, respectively.

## Methods

### Procedure

#### Sample 1

A retrospective cross-sectional analysis of all patients with well-differentiated NETs and liver metastases undergoing treatment at the University Medical Center Hamburg-Eppendorf (UKE) between 12/2008 and 04/2021 (*n* = 268) was conducted. We included patients who underwent radiologic evaluation of the liver and testing for GGT with 3 months or less in between. Recent surgical therapy of the tumor and interventional therapies targeting the liver had to be at least 6 months prior to the date of evaluation to rule out its potential confounding influence. Due to the considerable differences in tumor biology, course of disease and therapy, neuroendocrine carcinomas were not included in the analyses. Accordingly, all tumors were classified according to WHO 2022 classification [12]. Thus, all G3 neoplasms were well-differentiated NETs G3. Patients with neuroendocrine carcinoma (*n* = 41), extrahepatic cholestasis (*n* = 6) or missing data for either radiologic or laboratory findings (*n* = 117) were excluded. The final sample consisted of 104 patients.

#### Sample 2

To investigate longitudinal associations of GGT levels with radiographic response, a small sample of patients with well-differentiated NETs of the pancreas and liver metastases undergoing treatment with STZ/5FU in our center between 05/2005 and 12/2012 was analyzed. Radiographic response of liver metastases (regression or progression) based on RECIST 1.1 criteria and GGT testing within 3 months was available for *n* = 15 patients.

### Measures

Radiologic studies included contrast-enhanced computed tomography (CT) (*n* = 38), contrast-enhanced MRI (*n* = 30), and positron emission tomography (PET)-CT (Tracer: ^68^Ga-DOTA-TATE) (*n* = 36). Imaging analyses were conducted in consensus by two radiologists with three and thirteen years of experience in abdominal radiology, who were blinded regarding clinical data and laboratory markers. The liver was evaluated in axial, sagittal, and coronal planes in all available sequences and contrast phases. Since volumetry of individual metastases is not feasible particularly in livers with high tumor burden, metastatic load was categorized visually as a percent estimate of total liver volume (very low, <10%; low, 10–25%; moderate, 25–50%; high, 50–80%; and very high, >80%), as recommended by the ENETS Consensus Guidelines [13]. Good inter- and intraobserver agreement has been shown for this visual semi-quantitative method [14].

### Statistical analysis

Statistical analysis was performed using IBM SPSS Statistics for Mac (Version 25) and Excel for Mac (Version 16.65). For cross-sectional analyses, three linear regression analyses were conducted with GGT as independent variable and age, gender, and clinical parameters as dependent variables (Model 1: age, gender, primary tumor location, liver tumor burden, Model 2: age, gender, grading, liver tumor burden; Model 3: age, gender, cholestasis, liver tumor burden). Besides, a oneway ANOVA analysis was performed to test for differences in GGT levels between five groups separated by liver tumor burden (1: <10%, 2: 10–25%, 3: 25–50%, 4: 50–80%, 5: >80%). Post-hoc analyses were conducted with Bonferroni correction. Longitudinal analysis of our STZ/5FU cohort was conducted using Wilcoxon tests for paired data. The significance level was set at *p* < 0.050.

## Results

Sociodemographic data, therapy-relevant variables, radiographic and laboratory parameters of the analyzed cross-sectional sample are shown in Table 1.

**Table 1:** Characteristics of the cross-sectional sample (*n* = 104)

Age (years), *M* (SD)	62.3 ± 12.6
Gender, *n (%)*	
Female	43 (41.3)
Primary tumor, *n (%)*	
Small bowel	49 (47.1)
Pancreas	40 (38.5)
Lung	4 (3.8)
Rectum	2 (1.9)
Kidney	1 (1.0)
Unknown	8 (7.7)
Grading, *n (%)*	
G1	29 (27.9)
G2	58 (55.8)
G3	13 (12.5)
GGT elevated, *n (%)*	54 (51.9%)
Mean GGT [*U/l]*, *M* (SD)	117.2 ± 173.6
Liver tumor burden, *n (%)*	
<10 %	18 (17.3%)
10–25 %	32 (30.8%)
25–50 %	21 (20.2%)
50–80 %	27 (26.0%)
>80 %	6 (5.8%)
Cholestasis, *n (%)*	15 (14.4%)
Central metastasis, *n (%)*	31 (29.8%)
Single largest metastasis, *n (%)*	
<5 cm	63 (60.6%)
5–10 cm	33 (31.7%)
>10 cm	8 (7.7%)

*M* mean, *SD* standard deviation, *G* Grading, *GGT* gamma-glutamyltransferase

Multivariate linear regression analyses showed a significant association between GGT levels and liver tumor burden in the general study population (*p* < 0.001), controlling for age, gender, primary tumor location, and cholestasis, as shown in Table 2.

**Table 2:** Association of GGT and liver tumor burden by controlling age, gender, grading, primary tumor location, and cholestasis

GGT	*B*	Standard error	*p*
Model 1			
Age	1.1	1.2	0.356
Gender, female	−40.5	31.6	0.204
Primary tumor			
Small bowel	−105.9	56.0	0.061
Pancreas	−51.0	56.9	0.372
Other	−44.4	77.1	0.566
Liver tumor burden (%)	70.6	12.4	0.000
Model 2			
Age	2.0	1.3	0.125
Gender, female	−52.7	31.8	0.100
Grading			
2	33.0	35.1	0.349
3	89.4	54.2	0.102
Liver tumor burden (%)	61.0	13.6	0.000
Model 3			
Age	1.2	1.2	0.331
Gender, female	−42.7	30.9	0.169
Cholestasis	−25.2	41.9	0.550
Liver tumor burden (%)	73.8	12.5	0.000

Statistical analysis was performed via multivariate linear regression. Model 1: Reference category of Primary tumor: unknown primary; Model 2: Reference category of Grading: 1. Nagelkerke’s *R*^2^*:* Model 1 = 0.109; Model 2 = 0.311; Model 3 = 0.296

*GGT* gamma-glutamyltransferase

**Fig. 2:**
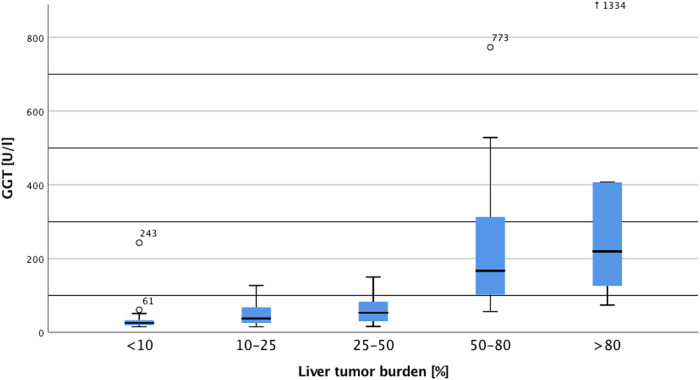
Levels of gamma-glutamyltransferase in relation to liver tumor burden. Notes: GGT = gamma-glutamyltransferase. Sample size: <10%: 18, 10–25%: 32, 25–50% 21, 50–80%: 27, >80%: 6

The prespecified groups based on liver tumor burden differed significantly in GGT levels (*p* < 0.001, Fig. 2). ANOVA analyses revealed that patients with high or very high tumor burden had increased GGT levels compared to patients with very low (both *p* < 0.001), low (both *p* < 0.001), and moderate tumor burden (*p* = 0.002 and *p* < 0.001, respectively). Between patients with very low, low, and moderate tumor burden, however, no differences in GGT levels were found (each *p* = 1.0). For predicting a liver tumor burden of >50%, GGT showed a sensitivity of 100% and a specificity of 70.4%. Positive and negative predictive value were 61.1% and 100%, respectively.

In our cohort of patients with pancreatic NET treated with STZ/5FU, regressive or progressive disease in the liver was observed in 4 and 11 cases, respectively (Table 3). In patients with regression, GGT levels decreased from a mean value of 271 U/l to 46 U/l. Due to the small sample size, Wilcoxon test showed no statistically significant difference (*p* = 0.067), yet four out of four patients showed a decline in GGT levels. In patients with progressive disease, mean GGT levels increased from 122 U/l to 337 U/l. This difference was statistically significant (*p* = 0.004) with 10 out of 11 patients showing an increase in GGT.

**Table 3:** Characteristics, outcomes, and laboratory findings of 15 patients with pancreatic NET under chemotherapy with Streptozotocin/5-Fluorouracil

Age in years, *M* (SD)	63 (13)
Gender, *n (%)*	
Female	10 (66.7)
Grading, *n* (%)	
G1	3 (20)
G2	10 (66.7)
G3	1 (6.7)
Hepatic progression, *n (%)*	11 (73.3)
GGT, start of therapy *[U/l]*	122 ± 89.9
GGT, time of progression *[U/l]*	337.3 ± 329.1
Follow-up time [months], *M* (SD)	16.3 (14.3)
Hepatic regression, *n (%)*	4 (26.7)
GGT, start of therapy *[U/l]*	271 ± 226.1
GGT, time of regression *[U/l]*	45.5 ± 14.3
Follow-up time in months, *M* (SD)	11.3 (6.7)

*M* mean, *SD* standard deviation, *G* Grading, *GGT* gamma-glutamyltransferase

## Discussion

In our study, we demonstrated for the first time an association between GGT and liver tumor burden in patients with NETs. Our data show that GGT elevation is common in those patients and is associated with a high liver tumor burden (>50%). Normal values imply low or moderate liver tumor burden, being of high negative predictive value. Longitudinal analysis of our STZ/5FU cohort showed, that GGT values change accordingly to the clinical course.

### Why do we find elevated GGT in patients with liver metastases?

The pathophysiology behind the rise in GGT levels in patients with liver metastasis has not been described yet. Interestingly, we did not observe a significant effect of radiologically detectable cholestasis on GGT levels. However, detectability of cholestasis on imaging depends on the imaging modality and is much lower on CT and PET-CT than on MRI. Furthermore, the rise of GGT could rather be driven by cholestasis in small bile ducts, undetectable in radiographic imaging. The tumor microenvironment of NETs has already been intensively researched. It could be shown that infiltrating immune cells mediate an immunosuppressive microenvironment [15, 16]. Therefore, peritumoral inflammation seems to be a less likely explanation for GGT elevations in NET liver metastases. A connection between GGT activity in the tumor’s membrane and hepatic tumor growth has been reported for melanoma cells [7]. As GGT expression in NET cells has not been investigated yet, increased cell turnover in large GGT-positive metastases would pose an explanation for the fact that GGT elevations are so common in our NET cohort. We therefore conducted a PCR analysis on GGT expression in four different NET cell lines compared to HEPG2-cells as a control sample. NET cells showed very low expression levels of GGT compared to HEPG2-cells (data not shown). Thus, NET cells do not seem to be the source of GGT elevations, it rather the surrounding liver tissue. Hence, NET metastases to other organs should not lead to elevated GGT.

### Association between GGT and liver tumor burden

Considering GGT as a biomarker for liver metastasis, no data for NETs were found. For renal cell carcinoma [9], ovarian cancer [6], and endometrial cancer [10], elevated serum GGT was shown to be of negative prognostic value in general. In patients with colorectal cancer, an initial decrease in GGT under therapy was associated with improved overall response and progression-free survival [17]. Yet, in the mentioned studies, there were no analyses on the presence or size of liver metastases. A correlation between GGT and liver tumor diameter and volume has so far only been demonstrated for hepatocellular carcinoma [8]. Liver metastases are common in a variety of solid neoplasms, particularly in GI cancer, and monitoring them is usually of great clinical importance. Our study demonstrates a significant association between GGT and liver tumor burden caused by metastases. This should also prompt further research in other oncologic entities. The clinical utility of GGT as a marker for liver tumor burden is favored by the fact that it is an established, easy-to-access and commonly performed test for various indications. It should be noted, however, that only half of our patients, whom all had liver metastases, showed an increase of GGT at the time of evaluation. Hence, GGT is not suited for ruling out liver metastases. If liver metastases are known though, normal GGT values might rule out a tumor burden >50%, according to our data. Greater clinical utility of GGT testing might, however, be achieved in serial testing over the course of treatment.

### Laboratory biomarkers for NETs

Due to the slow growth dynamics of NETs and consecutively long treatment and follow-up periods, there is a clinical need for therapy monitoring with laboratory markers. A whole range of biomarkers are known for NETs, although some are only applicable to specific entities [18]. Measurement of 5-hydroxyindoleacetic acid in either urine or plasma may be of use as a biomarker in functionally active NETs of the small intestine, yet it has not shown to be a reliable prognostic marker [19]. Chromogranin A (CgA) is a protein found in cells of neuroendocrine origin and has been the most widely used biomarker for neuroendocrine neoplasms in general to date [20]. It has shown to be predictive of disease-free survival after surgery as well as therapeutic response and is associated with a high liver tumor burden [21–23]. A recent study found that CgA is associated with disease progression in pancreatic NETs and predictive of negative outcome in patients with small intestine or cecum NETs, however, the results were limited to these subgroups [24]. Regarding its role as a follow-up parameter, a review and meta-analysis showed that it has sufficient accuracy, especially when baseline values are impaired [25] Other authors conclude that the sensitivity and specificity of CgA are insufficient for its use as a clinical biomarker [26]. For example, it has been shown that CgA is also elevated in chronic liver diseases such as cirrhosis, hepatitis and hepatocellular carcinoma [27]. Furthermore, not all NETs reliably express and secrete CgA, limiting its use in routine clinical practice [28]. NETest is a novel diagnostic tool based on mRNA detection in the patient’s blood [29]. Recent studies have shown it to be of high diagnostic accuracy and predictive of disease progression or stable disease, respectively [30]. In comparison to CgA, it was found to be far more accurate in predicting therapy response or progression free survival [31]. However, NETest is still not in routine clinical use and costs are estimated to be very high (3000–4000$/year). Like GGT, alkaline phosphatase (AP) is a widely used laboratory marker for cholestasis. Studies have shown it to be a negative predictor of survival in patients treated for NETs [32, 33]. Another recent study retrospectively analyzing 49 NET patients confirmed the negative prognostic value of AP, but it found no correlation between AP levels and the quantity or size of metastases [34]. Since AP elevation was detected in only one in three patients and no correlation with the disease extent was found, it may be less suitable as a laboratory follow-up marker for NET patients.

To date, no study has focused on the potential role of GGT for therapy monitoring in patients with neuroendocrine liver metastases. However, since GGT testing is often performed, clinicians tended to attribute a rise in GGT with hepatic progression. Hence, our study poses an evaluation of an until then common clinical practice. The results support this assumption, showing that there is indeed an association between GGT and liver tumor burden. It should be noted, though, that low or moderate liver tumor burden may not be detected by GGT testing. In patients with a low liver tumor burden, the serial testing of GGT, therefore, seems to be helpful only insofar as an increase in GGT can indicate progression of the disease. Normal values do not exclude progression, as in our analyses the GGT values only rise reliably above a tumor burden of 50%. Accordingly, serial GGT tests may be of less utility in these patients. However, from a clinical point of view, close surveillance is of greater importance in patients with high tumor burden, as these patients have a worse prognosis [35]. In case of a sudden increase in GGT, liver tissue damage due to other conditions should be considered. Change dynamics might help to distinguish between liver tissue damage or malignant progression, as GGT due to NET progression appeared to increase slowly and steadily, matching the clinical course of the patients.

When directly comparing GGT and the current standard CgA as biomarkers for neuroendocrine tumor disease, there are some important points to consider. Whereas CgA is of prognostic value for intra- and extra-hepatic disease, GGT has only been evaluated for liver tumor burden. Using CgA as a biomarker is only possible in tumors expressing and secreting CgA. In contrast, GGT is not dependent on tumor-specific features and might be of value in all different kinds of NETs with liver involvement. However, a larger study addressing this point is warranted. As GGT determination is part of routine laboratory diagnostic, results are often immediately available, whereas CgA determination is restricted to specialized laboratories and is often performed only once a week or even less frequently, thus causing a delay in response to changing values.

### Limitations and strengths

There were several limitations to this study. This was a single-center study with relatively small sample sizes, especially in the longitudinal cohort. Due to the retrospective design, no causal conclusions can be drawn from the data. In addition, retrospective studies carry the risk of selection bias. Patients usually had multiple GGT testing with matching radiographic evaluation. The time of evaluation was therefore chosen individually for each patient, avoiding confounding factors such as liver or biliary duct interventions, operations, or other causes for GGT increase. However, we did not collect data on patients’ concomitant diseases (e.g., diabetes, metabolic syndrome) or the use of medication, which might have confounded the results. In addition, as GGT is not a specific lab test, there may have been unknown confounders facilitating GGT elevations. Imaging evaluation was done on a visual scale, a method which has been established in similar studies but to which a certain degree of subjectivity is inherent. This approach was chosen because a very large number of liver metastases were present in our patient collective, so volumetry of each one would not have been feasible. However, since readers were blinded to clinical information and laboratory findings, the subjectivity of the method does not create any systematic error. Regarding the longitudinal analysis, which was conducted exploratively, no direct conclusions can be drawn for clinical practice due to the small sample size. However, to our knowledge, this study is the first to investigate GGT as a biomarker for clinical follow-up of liver metastases.

### Implications

The association between GGT elevations and liver metastases demonstrated in this study should raise physicians’ awareness of possible disease progression when detected in a routine examination. Further research in larger longitudinal series is required to assess the utility of GGT as a follow-up parameter. If confirmed in future studies, GGT can be implemented in clinical practice as a very cost-effective tool for therapy monitoring of liver metastases under systemic treatment for NETs to trigger radiographic evaluation, thereby allowing timely detection of disease progression and adaptation of therapy.

## References

[1] Riihimäki M, Hemminki A, Sundquist K, Sundquist J, Hemminki K (2016). The epidemiology of metastases in neuroendocrine tumors. Int. J. Cancer.

[2] Pavel M (2016). ENETS consensus guidelines update for the management of distant metastatic disease of intestinal, pancreatic, bronchial Neuroendocrine Neoplasms (NEN) and NEN of unknown primary site. Neuroendocrinology.

[3] Nemesanszky E, Lott JA (1985). Gamma-glutamyltransferase and its isoenzymes: progress and problems. Clin. Chem..

[4] Lum G, Gambino SR (1972). Serum gamma-glutamyl transpeptidase activity as an indicator of disease of liver, pancreas, or bone. Clin. Chem..

[5] Bijnens EM (2021). Serum gamma-glutamyl transferase, a marker of alcohol intake, is associated with telomere length and cardiometabolic risk in young adulthood. Sci. Rep..

[6] Grimm C (2013). Association of gamma-glutamyltransferase with severity of disease at diagnosis and prognosis of ovarian cancer. Br. J. Cancer.

[7] Obrador E (2002). gamma-Glutamyl transpeptidase overexpression increases metastatic growth of B16 melanoma cells in the mouse liver. Hepatology.

[8] Zhang LX, Lv Y, Xu AM, Wang HZ (2019). The prognostic significance of serum gamma-glutamyltransferase levels and AST/ALT in primary hepatic carcinoma. BMC Cancer.

[9] Hofbauer SL (2014). Pretherapeutic gamma-glutamyltransferase is an independent prognostic factor for patients with renal cell carcinoma. Br. J. Cancer.

[10] Seebacher V (2012). Prognostic significance of gamma-glutamyltransferase in patients with endometrial cancer: a multi-centre trial. Br. J. Cancer.

[11] Salazar R (2022). LBA45 Randomized open label phase III study comparing the efficacy and safety of everolimus followed by chemotherapy (CT) with streptozotocin (STZ)-5FU upon progression or the reverse sequence, in advanced progressive panNETs: the SEQTOR study (GETNE 1206). Ann. Oncol..

[12] Rindi G (2022). Overview of the 2022 WHO classification of neuroendocrine neoplasms. Endocr. Pathol..

[13] Pavel M (2012). ENETS Consensus Guidelines for the management of patients with liver and other distant metastases from neuroendocrine neoplasms of foregut, midgut, hindgut, and unknown primary. Neuroendocrinology.

[14] Zappa M (2017). Is visual radiological evaluation of liver tumour burden in patients with neuroendocrine tumours reproducible. Endocr. Connect..

[15] Cives M (2019). The tumor microenvironment in neuroendocrine tumors: biology and therapeutic implications. Neuroendocrinology.

[16] Vesely C (2022). Systematic evaluation of the immune environment of small intestinal neuroendocrine tumors. Clin. Cancer Res..

[17] Gong Z (2021). AKP and GGT level can provide an early prediction of first-line treatment efficacy in colorectal cancer patients with hepatic metastases. Biomark. Med..

[18] Oberg K (2017). ENETS consensus guidelines for standard of care in neuroendocrine tumours: biochemical markers. Neuroendocrinology.

[19] Zandee WT, Kamp K, van Adrichem RC, Feelders RA, de Herder WW (2016). Limited value for urinary 5-HIAA excretion as prognostic marker in gastrointestinal neuroendocrine tumours. Eur. J. Endocrinol..

[20] Nobels FR (1997). Chromogranin A as serum marker for neuroendocrine neoplasia: comparison with neuron-specific enolase and the alpha-subunit of glycoprotein hormones. J. Clin. Endocrinol. Metab..

[21] Shanahan MA (2016). Chromogranin A predicts survival for resected pancreatic neuroendocrine tumors. J. Surg. Res.

[22] Han X (2015). The value of serum chromogranin A as a predictor of tumor burden, therapeutic response, and nomogram-based survival in well-moderate nonfunctional pancreatic neuroendocrine tumors with liver metastases. Eur. J. Gastroenterol. Hepatol..

[23] Arnold R (2008). Plasma chromogranin A as marker for survival in patients with metastatic endocrine gastroenteropancreatic tumors. Clin. Gastroenterol. Hepatol..

[24] Fuksiewicz M (2018). Prognostic value of chromogranin A in patients with GET/NEN in the pancreas and the small intestine. Endocr. Connect.

[25] Rossi RE, Ciafardini C, Sciola V, Conte D, Massironi S (2018). Chromogranin A in the follow-up of gastroenteropancreatic neuroendocrine neoplasms: is it really game over? A systematic review and meta-analysis. Pancreas.

[26] Marotta V (2018). Chromogranin A as circulating marker for diagnosis and management of neuroendocrine neoplasms: more flaws than fame. Endocr. Relat. Cancer.

[27] Massironi S (2009). Chromogranin A levels in chronic liver disease and hepatocellular carcinoma. Dig. Liver Dis..

[28] Koenig A (2017). Clinicopathological hallmarks and biomarkers of colorectal neuroendocrine neoplasms. PLoS One.

[29] Modlin IM (2018). The NETest: the clinical utility of multigene blood analysis in the diagnosis and management of neuroendocrine tumors. Endocrinol. Metab. Clin. North Am..

[30] Liu E (2019). Assessment of NETest Clinical Utility in a U.S. registry-based study. Oncologist.

[31] Malczewska A (2020). The clinical applications of a multigene liquid biopsy (NETest) in neuroendocrine tumors. Adv. Med. Sci..

[32] Clancy TE (2006). Alkaline phosphatase predicts survival in patients with metastatic neuroendocrine tumors. Digest. Dis. Sci..

[33] Onesti JK (2016). Elevated alkaline phosphatase prior to transarterial chemoembolization for neuroendocrine tumors predicts worse outcomes. J. Gastrointest. Surg..

[34] Andriantsoa M (2017). An elevated serum alkaline phosphatase level in hepatic metastases of grade 1 and 2 gastrointestinal neuroendocrine tumors is unusual and of prognostic value. PLoS One.

[35] Rinke A (2017). Placebo-controlled, double-blind, prospective, randomized study on the effect of octreotide LAR in the control of tumor growth in Patients with Metastatic Neuroendocrine Midgut Tumors (PROMID): results of long-term survival. Neuroendocrinology.

